# Injectable hydrogel loaded with lysed OK-432 and doxorubicin for residual liver cancer after incomplete radiofrequency ablation

**DOI:** 10.1186/s12951-023-02170-0

**Published:** 2023-11-02

**Authors:** Yanyan Cao, Tao Sun, Bo Sun, Guilin Zhang, Jiayun Liu, Bin Liang, Chuansheng Zheng, Xuefeng Kan

**Affiliations:** 1 Department of Radiology, Union Hospital, Tongji Medical College, Huazhong University of Science and Technology, Wuhan, 430022 China; 2 Hubei Province Key Laboratory of Molecular Imaging, Wuhan, China

## Abstract

**Objective:**

To investigate the efficacy of an injectable hydrogel loaded with lysed OK-432 (lyOK-432) and doxorubicin (DOX) for residual liver cancer after incomplete radiofrequency ablation (iRFA) of hepatocellular carcinoma (HCC), and explore the underlying mechanism.

**Materials and Methods:**

The effect of OK-432 and lyOK-432 was compared in activating dendritic cells (DCs). RADA16-I (R) peptide was dissolved in a mixture of lyOK-432 (O) and DOX (D) to develop an ROD hydrogel. The characteristics of ROD hydrogel were evaluated. Tumor response and mice survival were measured after different treatments. The number of immune cells and cytokine levels were measured, and the activation of cGAS/STING/IFN-I signaling pathway in DC was evaluated both in vitro and in vivo.

**Results:**

LyOK-432 was more effective than OK-432 in promoting DC maturation and activating the IFN-I pathway. ROD was an injectable hydrogel for effectively loading lyOK-432 and DOX, and presented the controlled-release property. ROD treatment achieved the highest tumor necrosis rate (*p* < 0.001) and the longest survival time (*p* < 0.001) compared with the other therapies. The ROD group also displayed the highest percentages of DCs, CD4^+^ T cells and CD8^+^ T cells (*p* < 0.001), the lowest level of Treg cells (*p* < 0.001), and the highest expression levels of IFN-γ and TNF-α (*p* < 0.001) compared with the other groups. The expression levels of pSTING, pIRF3, and IFN-β in DCs were obviously higher after treatment of lyOK-432 in combination with DOX than the other therapies. The surviving mice in the ROD group showed a growth inhibition of rechallenged subcutaneous tumor.

**Conclusion:**

The novel ROD peptide hydrogel induced an antitumor immunity by activating the STING pathway, which was effective for treating residual liver cancer after iRFA of HCC.

**Supplementary Information:**

The online version contains supplementary material available at 10.1186/s12951-023-02170-0.

## Introduction

Liver cancer is the sixth most common cancer and the third leading cause of cancer-related deaths worldwide [1]. Thermal ablation, such as the prevalent radiofrequency ablation (RFA), is an important method for eradicating small (≤ 3 cm) lesions with minimal invasion [2]. However, RFA is usually limited in destroying the larger and irregular tumors, with peripheral residual tumor tissues alive [3, 4]. The residual viable cancer cells tend to be more malignant, leading to tumor recurrence and progression [4–6].

Immunotherapy is a promising treatment, but monotherapy with immune checkpoint inhibitors is efficacious in only approximately 15–20% of patients with hepatocellular carcinoma (HCC) [7]. The heavy tumor burden and “cold” tumor microenvironment are culpable for the unfavorable outcomes [7, 8]. Although incomplete RFA (iRFA) could reduce tumor volume and may arose antitumor immunity by releasing tumor-associated antigens, the immune response is probably overwhelmed by the deteriorative immunosuppressive microenvironment after iRFA treatment [9]. Therefore, inducing substantial improvement in antitumor immunity might be a rational strategy to enhance the antitumor efficiency.

OK-432, a penicillin-killed and lyophilized preparation of a low-virulence strain (Su) of *Streptococcus pyogenes* (group A), has been used as an immunomodulator for the therapy of various cancers [10, 11]. As a foreign antigen, OK-432 plays important roles in the recruitment and maturation of the most potent antigen-presenting dendritic cells (DCs), which are rare within HCC tissues but associated with the prognosis of patients [12, 13]. However, the therapeutic efficacy of OK-432 for HCC needs improvement [14, 15]. Up till now, the inhibitory effect of lysed OK-432 (lyOK-432) on cancer and the relevant mechanism have not been reported. Theoretically, the lyOK-432 can directly provide more foreign antigens and bacteria-derived DNA containing unmethylated CpG motifs [16]. The unmethylated CpG motifs can be perceived by the DNA sensor (cyclic guanosine phospho-adenosine synthase, cGAS) in DCs, thereby activating the downstream protein of stimulator of interferon genes (STING), and secreting the cytokine of type I interferon (IFN-I), which is supposed to improve the antitumor immunity [17]. However, the effect of lyOK-432 on HCC after iRFA warrants further investigation.

In addition to direct killing cancer cells, chemotherapy also modulates tumor immunobiology. For example, standard chemotherapy produces immunosuppressive effects by killing various immune cells [18]. However, lower dose of doxorubicin (DOX) is capable of decreasing suppressive immune cells, such as MDSCs and Treg cells [8, 19]. Moreover, DOX intercalates into DNA of cancer cell and triggers a DNA damage response [20]. The damaged DNA probably could serve as an activator of the cGAS sensor, thus enhancing the secretion of IFN-I in DCs. However, as we known, DOX monotherapy is insufficient to destroy liver cancer.

Given these limitations, in this study, we tried to combine lyOK-432 with DOX to enhance the effect of RFA on HCC. Besides, an injectable hydrogel, RADA16-I, was adopted for loading and controlled-releasing of lyOK-432 and DOX (ROD) in the present study. RADA16-I is a simple, biocompatible, and amphiphilic synthetic peptide with the RADARADARADARADA sequence. RADA16-I can self-assemble into a peptide nanofiber hydrogel, which is well-suited for encapsulating a broad range of hydrophilic and hydrophobic agents [21]. The present study was designed to investigate the synergistic effect of ROD for treating residual liver cancer after iRFA of HCC and explore its underlying mechanisms.

## Materials and methods

This study was approved by the Ethics Committee of Tongji Medical College, Huazhong University of Science and Technology ([2021] IACUC Number: 2978), Wuhan, China. RADA16-I (Ac-RADARADARADARADA-NH_2_) was synthesized by Bankpeptide Ltd. (Hefei, China). DOX and OK-432 were purchased from the Department of Pharmacy, Union Hospital, Huazhong University of Science and Technology.

### Preparation of lyOK-432

LyOK-432 was dissolved in 0.9% NaCl solution (1 KE/mL) at room temperature, and then subjected to more than 3 freeze–thaw cycles. The comparison between lyOK-432 and OK-432 was made in terms of maturity of DCs after intervention, as indicated by the expression of surface markers using flow cytometry and the level of cytokine secretion using ELISA. Additionally, the protein expressions of pSTING and pIRF3 were measured by Western blotting, and IFN-β was determined by qPCR and ELISA.

### Hydrogel synthesis

In Ref [21]. LyOK-432 (1 KE/ml) and DOX (2 mg/ml) were dissolved in 0.9% NaCl solution at room temperature, and RADA16-I (R) peptide (10 mg) was dissolved in a mixture of DOX and/or lyOK-432 (1 ml) and repeated pipetting was conducted for complete dissolution. These solutions were then kept at 4 ℃ under sterile conditions overnight to allow for the formation of ROD (R + lyOK-432 + DOX), RO (R + lyOK-432), RD (R + DOX), and R hydrogels.

### Transmission electron microscopy (TEM) imaging and rheological analysis

A hydrogel sample (5 μL) was drop-cast onto the clean surface of a copper grid and dried using bibulous paper after dilution with ultrapure water at a ratio of 1:20. Next, the samples were stained with phosphotungstic acid (5 μL) for 30 s and dried with bibulous paper. TEM (Titan G2 60–300; FEI Company, OR) was then conducted to record the morphology of these hydrogels. A rheometer (DHR-2, TA, Instruments, New Castle, DE, USA) was used to evaluate the rheological characteristics of the ROD hydrogel (1%, w/w). The storage modulus (G’) and loss modulus (G’’) were measured while maintaining the strain at 0.1% at a frequency of 0.1–100 rad/s. A constant frequency of 1 rad/s was used to detect the thixotropic properties. A low constant strain of 0.1% for 200 s, a higher strain of 40%, and a recovery to a constant strain of 0.1% were performed successively to evaluate the ROD hydrogel.

### Drug release and gel degradation

ROD or RD hydrogel (0.5 mL) was added to a centrifuge tube (1.5 mL), and then 0.9% NaCl (1 mL) was infused with or without proteinase K (5 U/mL) at 37 ℃. The top buffer was extracted at the indicated times to measure the concentration of DOX, and the remaining mass was accurately weighed. Then, an equal volume of fresh buffer was added. The intensity of DOX fluorescence in the buffer was evaluated on a fluorospectrophotometer (F97XP15007; Shanghai Lengguang Technology Co., Ltd., China) at an excitation wavelength of 500 nm and an emission wavelength of 550 nm.

### In vitro DC maturation and cell viability

Immuture DCs (imDCs) were derived from C57BL/6 mice. Briefly, murine bone marrow was initially cultured at 0.5 × 10^6^ cells/mL in RPMI 1640 with 10% fetal bovine serum containing 20 ng/mL granulocyte–macrophage colony-stimulating factor (GM-CSF) (R&D Systems, MN, USA). After 7 days of culture, the bone marrow-derived dendritic cells (BMDCs, or imDCs) were generated.

Hep1-6 cells (1.5 × 10^5^ cells/well) were seeded onto a 96-well plate and cultured at 42 ℃ for 24 h upon addition of OD, O, D, and PBS to each well. After incubation for 24 h at 37 ℃, the cell supernatant was harvested.

ImDCs were seeded into 24-well plates (1.5 × 10^5^ cells/well), and the hep 1–6 cell supernatant was centrifuged to obtain debris, which was then added to each well. After incubation for 24 h, the cellular surface markers of CD80 and CD86 were detected using flow cytometry.

The CD8^+^ T cells were derived from a transgenic mouse expressing a tumor antigen-specific TCR from Hep 1–6. The isolated CD8^+^ T cells were co-incubated with hep 1–6 specialized mature DCs for 48 h, which were isolated using magnetic beads. After isolating the stimulated CD8^+^ T cells by Isolation Kit (EasySep, BD), hep 1–6 cells (2 × 10^4^) were plated into each well of a 96-well plate and co-incubated with CD8^+^ T cells. The cell viability of hep 1–6 was evaluated 12 h later using CCK-8 assay.

### CGAS/STING/IFN-I expression

The protein expression of pSTING and pIRF3 was measured by Western blotting, as previously described [22]. Briefly, proteins from cells and tumor tissues were extracted, separated, put onto polyvinylidene difluoride membranes, then blocked and incubated with antibodies against cGAS (D3O8O), pSTING (Ser366), and pIRF3 (29047S) (Cell Signaling Technology, MA, USA). The luminescence intensity of the bands was detected by using an enhanced ECL kit (Thermo Fisher Scientific Inc., USA). The expression of IFN-β was determined by employing qPCR (Invitrogen) and an ELISA kit (Dakewe Biotech, China) according to the manufacturer’s instructions. For qPCR, the total RNA of the cells and tumor tissues were extracted, homogenized, and treated with DNase. Then 50 ng of total RNA was used for each qPCR reaction. Data were analyzed using the ΔΔCT method and normalized to β-actin (primers: PF: CAGCTCCAAGAAAGGACGACC; PR: GGCAGTGTAACTCTTCTGCAT).

### Biodistribution imaging

Hep1-6 cells (1 × 10^6^) were subcutaneously injected into the right flank of female C57BL/6 mice (4–6 weeks age). When tumors grew to ∼100 mm^3^, mice in the three groups were intratumorally injected with 100 μL ROD, RD and DOX (n = 3), respectively. DOX was administered at the same dosage as that of ROD. The fluorescence signals were measured at the indicated time points by using a small animal imaging system IVIS Kinetic (excitation wavelength: 465 nm; emission wavelength: DsRed).

### HCC model in situ, iRFA, and treatment

Medium liver lobes of female C57BL/6 mice (4–6 weeks age) were injected with Hep1-6 cells (1 × 10^6^). Tumor volume was measured by magnetic resonance imaging (MRI, T2WI) and calipers, and calculated according to the equation: (Length × Wide^2^)/2. When the tumors grew to ∼100 mm^3^, the mice were randomly divided into different groups (the ROD, RO, RD, OD, O, D, R, and NS groups) and subjected to iRFA. Briefly, a monopolar radiofrequency needle was used to penetrate a small incision into the lower left quadrant of the tumor. Five Watts of ablation power was pressed and held for 30 s, and then the residual tumor tissue was intratumorally-injected with ROD, RO, RD, OD, O, D, R, and NS, respectively. Then, the abdomen of the mice was closed by suturing, and feeding continued for 14 days.

### Histological and survival analysis

Two weeks after treatment, the mice were sacrificed, and the tumor tissues were fixed with 4% paraformaldehyde and paraffin-embedded. Three-micrometer-thick sections were stained with hematoxylin–eosin (HE), TUNEL kit, Ki-67, CD4, CD8, Foxp3, and CD11c for histological analysis. The TUNEL assay kit (Roche, Shanghai, China) and monoclonal antibodies against Ki-67 (Sigma Sp6), CD4^+^ T (Servicebio, Wuhan, China), CD8^+^ T (Servicebio, Wuhan, China), Foxp3^+^ Treg (Servicebio, Wuhan, China), and CD11c^+^ DC (Servicebio, Wuhan, China) were used. In addition, the survival time of the mice was recorded.

### Flow cytometry

Seven days after treatment, the mice were sacrificed, and the tumor tissues were collected and digested in a buffer containing 0.2% collagenase A, 0.002% DNAase, and 0.01% HAase at 37 ℃ for 30 min. After grounding and filtering, the harvested single cells were collected. Antibodies against FSV780, CD45, CD3, CD4, CD8, CD25, CD11b, CD11c, MHCII, CD80, CD86, Gr-1, and CD206 were purchased from BD Biosciences. Blocking antibodies included ICH1077, ICH1042, and ICH1043 (all purchased from ichorbio, USA), respectively against DCs, CD4^+^ T cells, and CD8^+^ T cells. To stain intracellular IFN-γ and TNF-α, cells were stimulated with PMA (50 ng/mL) and ionomycin (500 ng/mL) for 4 h. Subsequently, the cells were incubated with brefeldin A (10 µg/mL) for 1 h. Following this, the cells were permeabilized using a Foxp3 Fixation and Permeabilization Kit (eBioscience) and stained with IFN-γ, TNF-α according to the manufacturer's protocols. For intracellular cytokine staining, the cells were permeabilized using a FoxP3 Fixation and Permeabilization Kit and stained for Foxp3. Subsequently, the stained cells were analyzed by flow cytometry (BD X20). The data were analyzed using Flow Jo software (Tree Star, Ashland, OR).

### Cytokine measurement

The peripheral blood and tumor of mice were collected. Peripheral blood was coagulated for 30 min, and tumor was dispersed in PBS. After centrifuge for 5 min at 1000 g, the supernate was collected. The expression levels of IFN-β, IFN-γ, and TNF-α in the supernate were measured by utilizing ELISA kits (Dakewe Biotech, China) according to the manufacturer’ protocols.

### Safety assessment

Body weight was measured at the indicated times. The major organs (heart, liver, spleen, lung, and kidney) were HE stained. Blood urea nitrogen (BUN), creatinine, aspartate aminotransferase (AST), and alanine aminotransferase (ALT) levels were measured to assess renal and hepatic functions.

### Statistical analysis

Statistical analysis was performed by using the SPSS software (SPSS, version 24.0, Chicago, IL, USA). Image-Pro Plus 6.0 (Media Cybernetics, Rockville, MD, USA) was used to analyze the tumor sections. Data are presented as the mean ± standard deviation. The measurement data among different groups were analyzed using One-Way ANOVA or Student’s t-test. Survival curves were plotted according to the Kaplan–Meier method, and analyzed using the log-rank test. A *p* < 0.05 was considered to be statistically significant (*p < 0.05, **p < 0.01, ***p < 0.001).

## Results

### LyOK-432 had a stronger promoting effect on DC maturation

As shown in Additional file Materials (Additional file 1: Figure S1), the rate of mature DCs (CD80^+^CD86^+^ DC) was significantly higher in the lyOK432 group than in the OK-432 and lipopolysaccharide (LPS) groups (*p* < 0.001 and *p* < 0.001, respectively). Additionally, DCs in the lyOK-432 group had the highest expression levels of IL-6 and IL-12 compared with the other three groups (*p* < 0.001). These results indicated that lyOK-432 promoted DC maturation more efficiently than non-lysed OK-432.

### Preparation and characterization of ROD

**Fig. 1:**
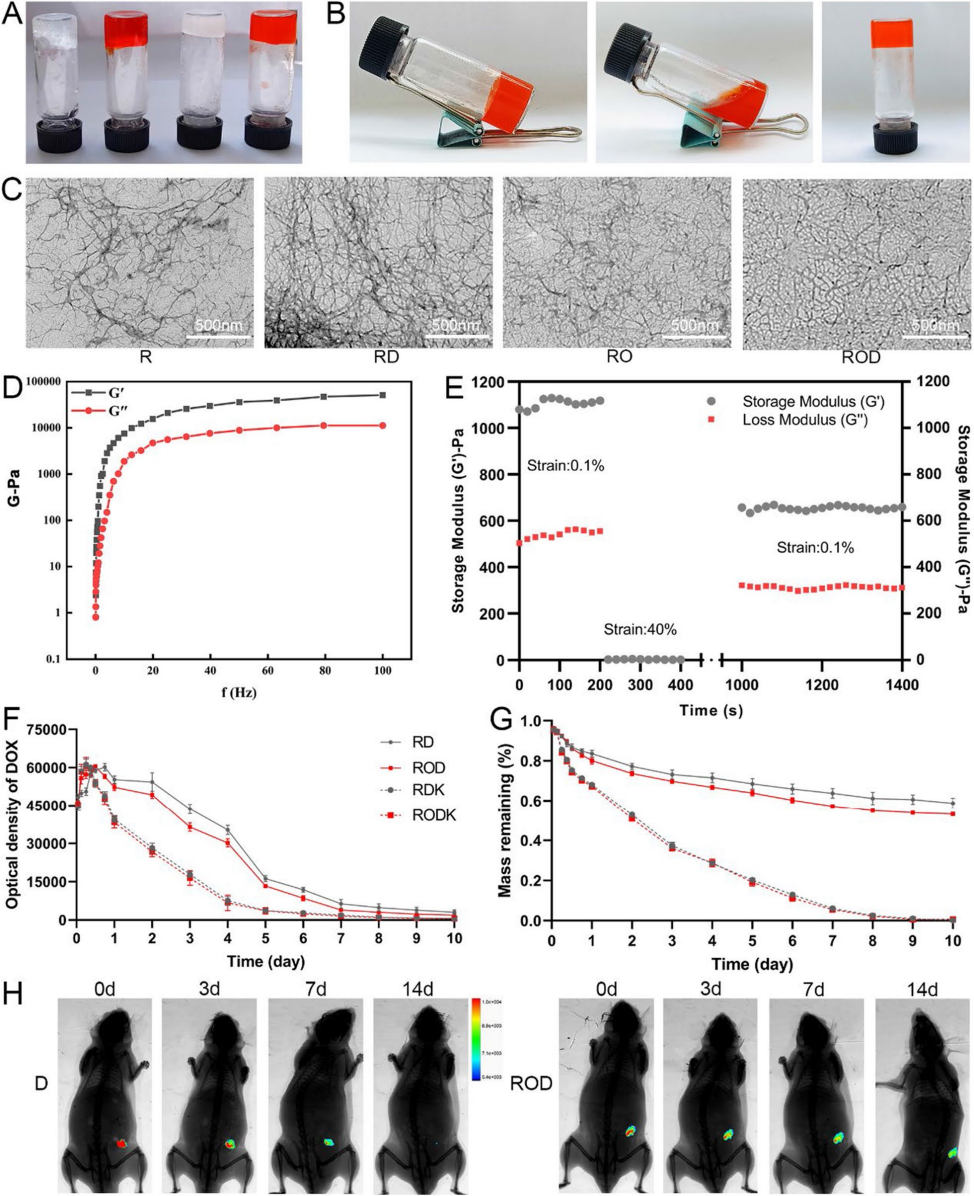
Preparation and characterization of ROD hydrogel. **A** R, RD, RO, and ROD hydrogel gelated for 7 days. **B** Thixotropic property of the ROD hydrogel. **C** TEM images of R, RD, RO, and ROD hydrogel, showing that these hydrogels self-assembled into networks of interwoven nanofibers. **D** Frequency sweep rheological analysis of the ROD hydrogel, suggesting the formation of a stable hydrogel. **E** Step-strain time-dependent rheological analysis of the ROD hydrogel, indicating ROD had a good rheological nature of for injection. Measurements were performed at a fixed angular frequency of 1 rad/s. **F** The release profiles of RD and ROD hydrogel-loaded DOX in the presence or absence of proteinase K. **G** The measurement of the weight of RD and ROD hydrogels in the presence or absence of proteinase K. **H** The release profile of DOX in ROD hydrogel and DOX solution after intratumor injection in the subcutaneous Hep1-6 tumor (n = 3), suggesting a good controlled-release nature of ROD hydrogel. ROD, RADA16-I peptide hydrogel loaded with lyOK-432 and doxorubicin; RO, RADA16-I peptide hydrogel loaded with lyOK-432; RD, RADA16-I peptide hydrogel loaded with doxorubicin; R, RADA16-I peptide hydrogel; *DOX D* doxorubicin, *TEM* transmission electron microscopy

The synthesis of ROD, RO, RD, and R was described in the Materials and Methods section. The RADA16-I peptide hydrogel was reported a good platform for loading a broad range of hydrophilic and hydrophobic components [21]. The four kinds of peptide hydrogels in this study were also successfully formed (Fig. 1A). To determine the stable loading capacity of ROD, we first changed the pH condition and results showed that the ROD peptide gelated at pH of 4.5–7.5, but did not form at 8.5 (Additional file 1: Figure S2), indicating the gelation capacity under physiological conditions and in tumor tissue. In addition, after repeated vigorous shaking, the ROD hydrogel became an injectable solution, indicating its thixotropic character (Fig. 1B).

TEM was used to evaluate the size and morphology of the ROD hydrogel. As shown in Fig. 1C, the ROD hydrogel self-assembled into networks of interwoven nanofibers. The rheological characteristics of the ROD hydrogel was evaluated in terms of the storage modulus (G’) and loss modulus (G’’). Figure 1D shows that G’ and G’’ were less dependent on the frequency (0.1–100 rad/s) as the strain constant increased, which suggested the formation of a stable hydrogel. In addition, a time-dependent step-strain rheological experiment was conducted to evaluate the injectability of the ROD hydrogel. During this process, a low constant strain of 0.1%, a higher strain of 40%, and a recovery strain of 0.1% were tested and recorded. Figure 1E shows that the ROD hydrogel virtually restored its original strength at 1000 s after withdrawal of the larger strain, which indicated that the ROD possessed a good rheological nature for injection.

### Drug release in vitro and in vivo

The release of DOX from the ROD hydrogel, with or without proteinase K, was measured in vitro using a fluorescence spectrophotometer. As shown in Fig. 1F, the optical density increased in the first 18 h, but gradually decreased thereafter in the four groups and was practically undetectable 10 days later. In addition, the optical density of DOX dropped steeply in the presence of proteinase K, suggesting an increased release of DOX from the hydrogel. On the other hand, the remaining peptide backbone of the ROD hydrogel was weighed. The results (Fig. 1G) showed that 53.5% of the ROD hydrogel remained after 10 days whereas it was completely digested with proteinase K, which suggested an excellent biodegradability of this hydrogel.

A subcutaneous Hep1-6 tumor model was established to detect drug release in vivo (Fig. 1H). After the tumor volume reached ∼100 mm^3^, the fluorescence intensity was high after intratumoral injection of 100 μL ROD or DOX on day 0. However, the fluorescence signal in the DOX group became weaker 3 and 7 days later, and was almost undetectable on the 14th day. In contrast, the fluorescence intensity after ROD injection was stronger on days 3 and 7, and the signal could still be detected 14 days later. Additionally, the fluorescence signal in the RD group was comparable to that of the ROD group, indicating that the incorporation of lyOK-432 did not alter the release profile of DOX in the hydrogel (Additional file 1: Figure S3). Collectively, these results suggested that ROD had a controlled-release nature.

### Antitumor effect of ROD

**Fig. 2:**
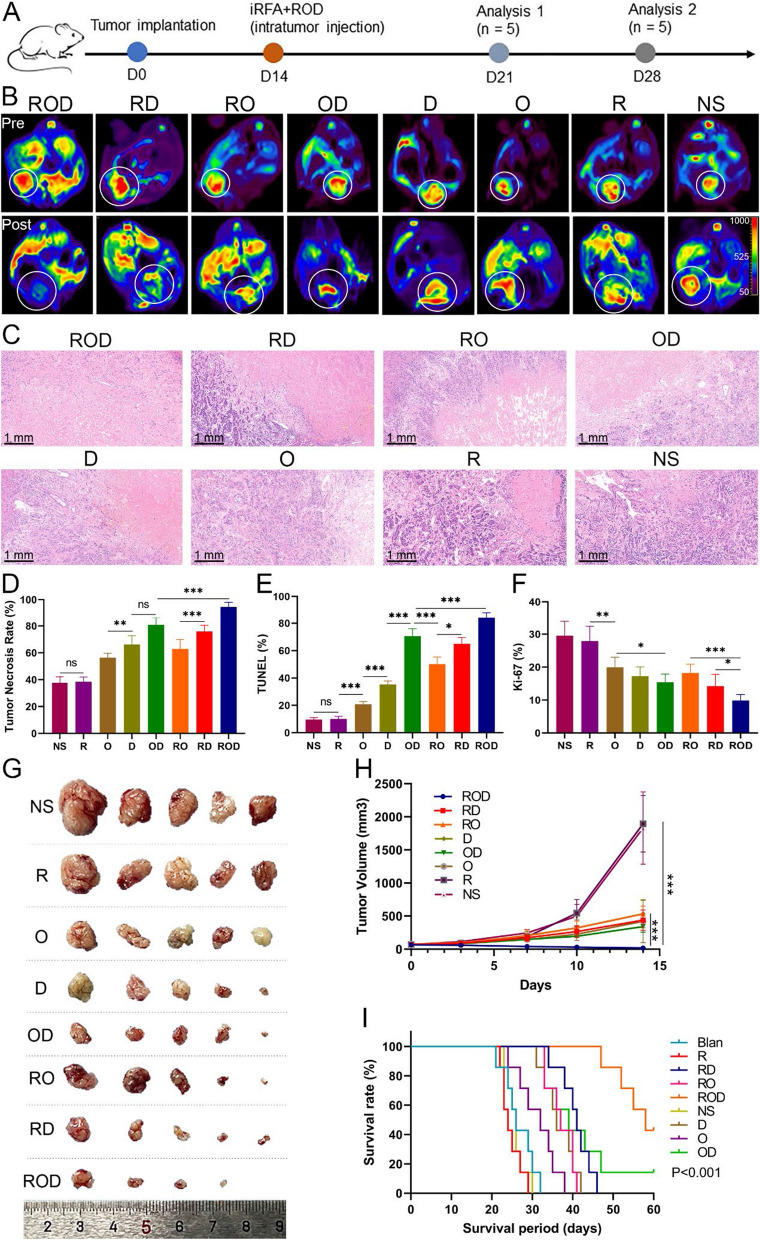
Antitumor effect of ROD hydrogel (n = 5). **A** Treatment schedule of mice bearing HCC. Analysis 1 refers to the evaluation of antitumor immunity, and analysis 2 refers to the evaluation of tumor treatment efficacy. **B** DWI images exhibiting the residual tumor tissues 14 days after treatments. **C** Representative HE-stained tumor sections (×10 magnification). **D**–**F** Quantitative analysis of tumor necrosis, apoptosis, and proliferation on the basis of HE staining, TUNEL assay, and Ki-67 staining. These results indicated the optimal antitumor effect was achieved in ROD group. **G** Photograph of dissected tumor samples. **H** Measurement of tumor volumes at the indicated times. Tumor progression was significantly inhibited in ROD group. **I** Kaplan–Meier survival curves show that the survival time of mice was significantly longer in the ROD group than in other groups (n = 7). *DWI* diffusion weighted imaging, *NS* normal saline; O, OK-432. *Ns* not significant; *p < 0.05; **p < 0.01; ***p < 0.001

To explore the anticancer effect of ROD hydrogel, a mouse model of orthotopic HCC was established. After iRFA, the residual viable tumor tissues in different groups were intratumorally injected with ROD, RO, RD, OD, O, D, R, and NS, respectively (Additional file 1: Figure S4). Tumor response was evaluated 14 days after treatment. As shown by the DWI images in Fig. 2B, the residual tumor tissues in the ROD group were rarely seen, while the signals of viable tumors were conspicuously stronger in other groups, especially in the NS and blank groups. In addition, the tumor necrosis rate was calculated according to HE staining, as previously described [23]. Results showed that the necrosis rate was 94.6% ± 1.5% in the ROD group, which was significantly higher than in the other 7 groups (75.9% ± 2.0% in the RD group, 62.9% ± 3.2% in the RO group, 80.8% ± 2.5% in the OD group, 66.3% ± 2.9% in the D group, 56.5% ± 1.4% in the O group, 38.4% ± 1.6% in the R group, and 37.6% ± 2.0% in the NS group, respectively; *p* < 0.001, Fig. 2C, D). TUNEL yield the highest rate of positive staining, while Ki-67 produced the lowest rate in the ROD group (*p* < 0.001, Fig. 2E, F, and Additional file 1: Figure S5), the findings being consistent with the results of the DWI imaging and HE staining. The tumor volume 14 days after treatment was also significantly smaller in the ROD group than in the other groups (*p* < 0.05, Fig. 2G, H).

Furthermore, survival analysis was conducted for the iRFA-treated mice in the 8 groups (Fig. 2I). Three mice (37.5%) in the ROD group survived for more than 60 days, which was significantly longer than the median survival time in the RD group (41 days), RO group (36 days), OD group (39 days), D group (36 days), O group (32 days), R group (24 days), NS group (26 days), and blank groups (26 days) (*p* < 0.001). Collectively, these results demonstrated that ROD treatment achieved a potent synergistic antitumor effect against liver cancer after iRFA therapy.

### ROD enhanced in vivo antitumor immunity

**Fig. 3:**
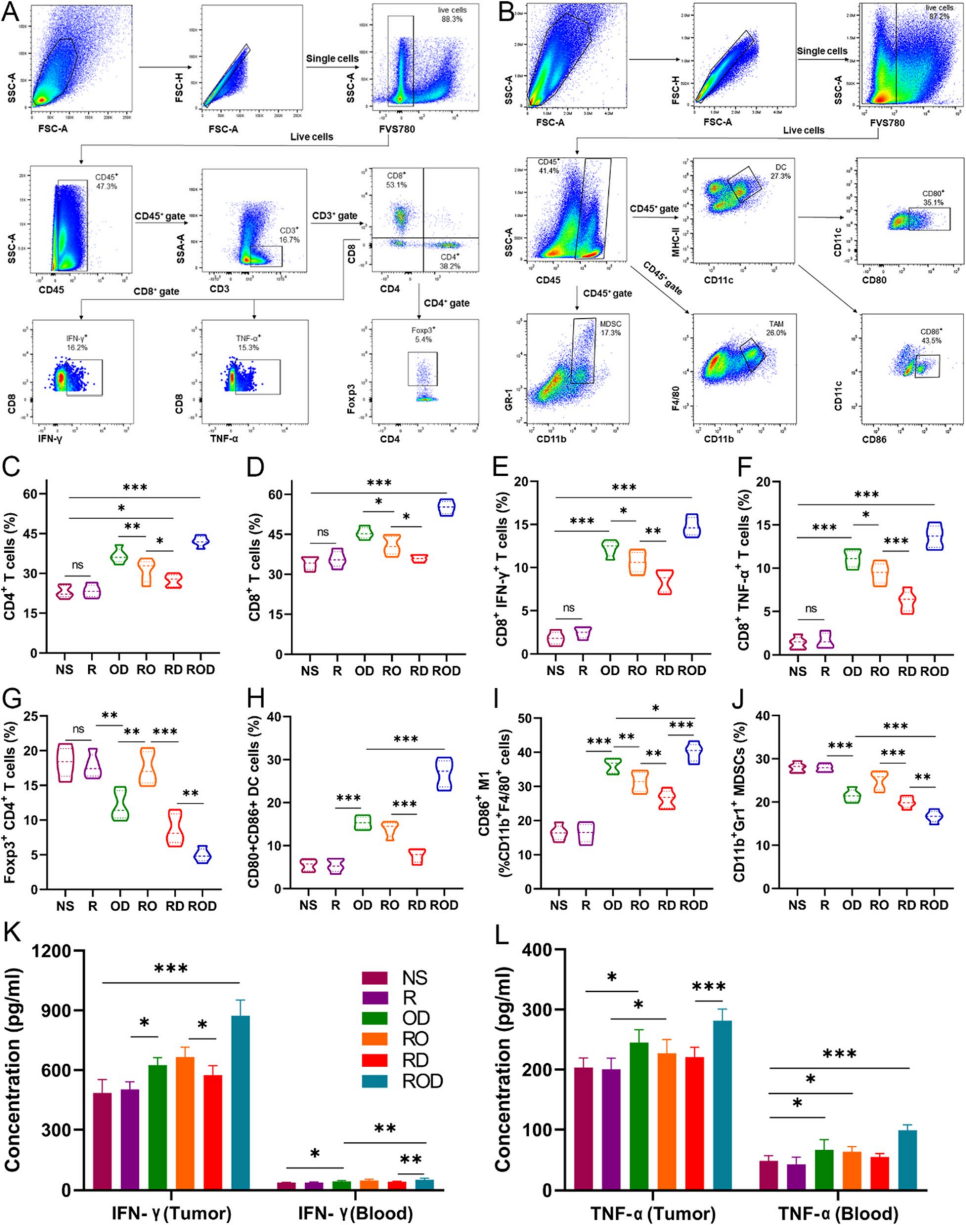
Enhanced antitumor immunity after ROD therapy (n = 5). **A**, **B** Representative dot plots of the morphological characteristics (SSC vs FSC) of tumors subjected to ROD. Schematic illustration of gating: Tumor-infiltrating lymphocytes were stained with corresponding antibodies for flow cytometric analysis, including CD4^+^ T, CD8^+^ T, Treg cells, DCs, TAM, and MDSCs. **C**–**J** Quantitative analysis of the percentages of CD4^+^ T, CD8^+^ T, Foxp3^+^CD4^+^ T, DCs, M1, and MDSCs in tumors 7 days after treatments. **K**, **L** Expressions of IFN-γ and TNF-α in tumor tissues and blood. *DC* dendritic cell, *TAM* tumor-associated macrophage, *MDSCs* myeloid-derived suppressor cell, *IFN* interferon, *TNF* tumor necrosis factor. *Ns* not significant; *p < 0.05; **p < 0.01; ***p < 0.001

To identify the immunological factors that impact therapeutic efficacy after iRFA, tumor-infiltrating lymphocytes (TILs) were evaluated after treatments, since previous studies indicated that DOX and OK-432 could trigger antitumor immunity [12, 19]. The tumor tissues were harvested at 7 days after treatments, and the immune cells were counted by flow cytometry. As presented in Fig. 3A–J, the results revealed that the ROD group had the highest levels of CD4^+^, CD8^+^ T cells and M1 polarized tumor-associated macrophages (M1-TAMs), while the R and NS groups had the lower counts (*p* < 0.001). The results of TAM and M2-TAM were shown in Additional file 1: Figure S6. Moreover, the proportions of CD4^+^ T cells, CD8^+^ T cells, and M1-TAM were also significantly higher in the RO and RD groups than in the R and NS groups (*p* < 0.001). In contrast, the proportion of Tregs and myeloid-derived suppressor cells (MDSCs) in the ROD group was significantly decreased compared with other groups (*p* < 0.01, Fig. 3G, J), and was significantly increased in the R and NS groups when compared with the blank controls (*p* < 0.001). Additionally, the levels of IFN-γ and TNF-α in tumor tissues and blood were determined by flowcytometry and ELISA (Fig. 3E, F, K, L). The results showed that these cytokines were all remarkably increased in the ROD group (*p* < 0.001), but were mildly elevated and significantly higher in the RO, RD, and OD groups compared with the NS and blank controls (*p* < 0.05). Immunohistochemical staining of TILs further demonstrated the findings (Additional file 1: Figure S7). However, the clearance of CD4^+^ T cells, CD8^+^ T cells, or both with antibodies remarkably impaired the antitumor efficacy of the ROD hydrogel (data not shown). Taken together, these results demonstrated that the ROD hydrogel efficiently improved the infiltration, accumulation, and function of antitumor immune cells.

### Improved antitumor immunity via enhanced cGAS/STING pathway in DCs

To further understand the mechanism of enhanced antitumor immune responses, DCs were measured by flow cytometry, which is the potent antigen presenting cells for activating the antitumor immune cells (Fig. 3A, H). The ROD group displayed the highest level of mature CD80^+^CD86^+^ DCs (mDC) compared with the other groups (*p* < 0.001). The proportion of mDCs in the RO, RD, and OD groups was significantly higher than in the NS group (*p* < 0.05). Immunohistochemical staining also confirmed these results (Additional file 1: Figure S7). However, blocking the DCs with antibody in the ROD group lower the tumor necrosis rate, which comparable to that of the RD and OD group (data not shown). Collectively, these results indicated that ROD, RO, RD, and OD treatment promoted DC maturation, followed by enhanced antigen presentation and immune cell recruitment in the residual liver cancer tissues 7 days after iRFA.

**Fig. 4:**
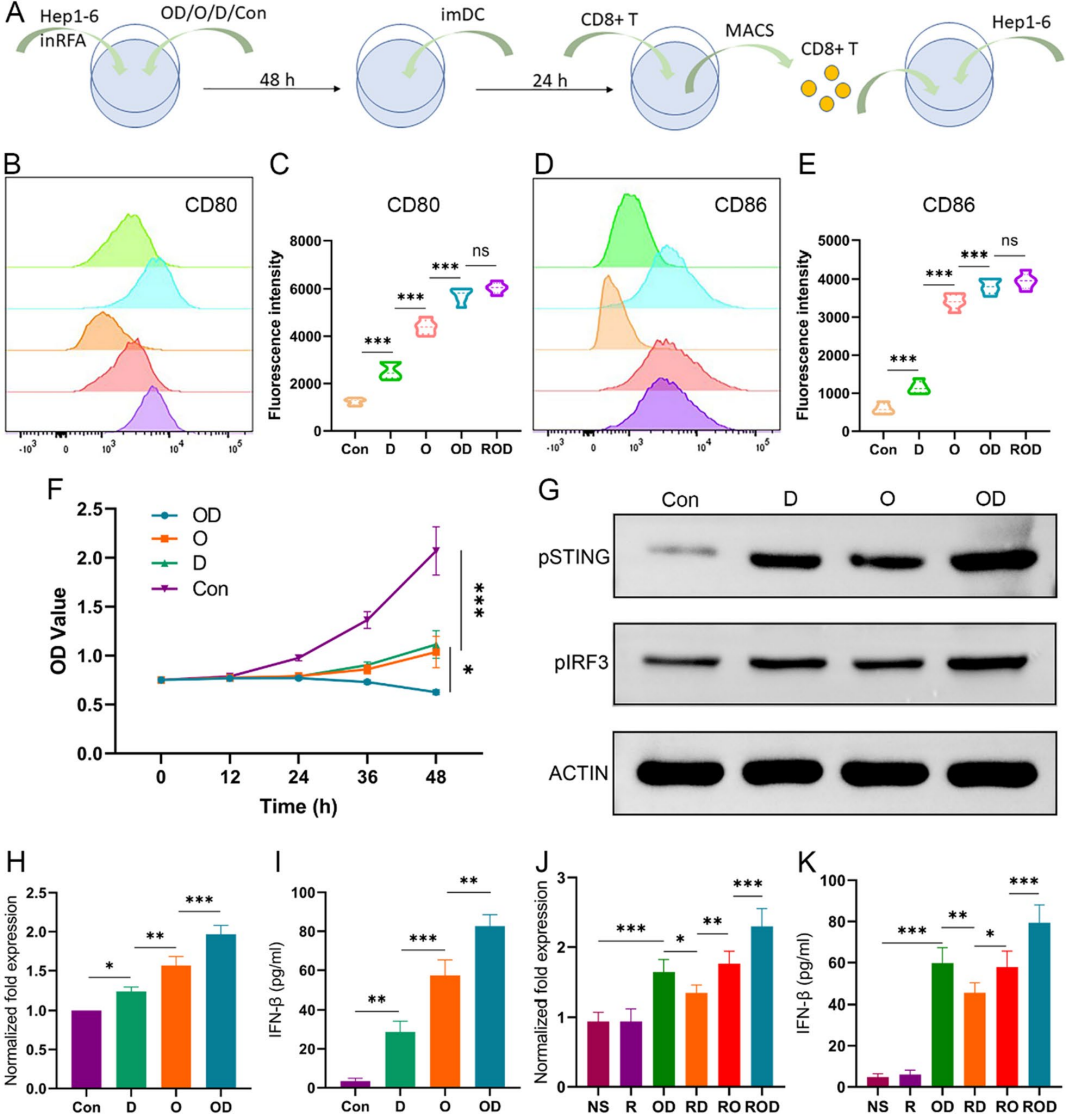
LyOK-432 in combination with DOX activated cGAS/STING pathway in DCs (n = 3). **A** In vitro illustration of study schedule for co-culture of Hep 1–6 cells, imDC, and CD8^+^ T cells after management of iRFA and OD, O, D, or control. (**B**–**E**) Quantitative analysis of the surface makers of mature DCs (CD80^+^ CD86^+^ DC). The expression level of CD80 and CD86 was highest in ROD/OD group. **F** The cytotoxicity of CD8^+^ T cells induced in vitro against Hep1-6 cells in the four groups as revealed by CCK-8 assay. The cancer cells were significantly suppressed after OD management. **G** Western blotting showed that the protein expression of phosphorylated STING and IRF3 was higher in OD group than in the other groups. **H**, **I** The IFN-β expression measured by qPCR and ELISA after different in vitro treatments. **J**, **K** The IFN-β expression in tumor tissues measured by qPCR and ELISA after different in vivo treatments (n = 5). cGAS, cyclic guanosine phospho-adenosine synthase; STING, stimulator of interferon genes; MACS, magnetic cell sorting. *p < 0.05; **p < 0.01; ***p < 0.001

Previous studies have indicated that OK-432, as a foreign antigen, could directly recruit DCs and promote their maturation, while DOX probably activate DCs along other pathways [12, 20]. Based on these hypotheses, we investigated the synergistic effect of ROD by examining the cGAS/STING/IFN-I signaling pathway in DCs both in vitro and in vivo. In in vitro experiments, Hep1-6 cells received iRFA management and ROD, OD, O, D, or left untreated (blank controls), and co-cultured with imDCs after 48 h (Fig. 4A). The surface markers of CD80^+^CD86^+^ mDCs were determined by flow cytometry. As shown in Fig. 4B–E, the proportion of mDC significantly increased after ROD and OD treatment (*p* < 0.01). In addition, hep1-6 cells were cocultured with CD8^+^ T cells after co-incubated with hep 1–6 specialized mature DCs. The results of CCK-8 showed a suppressed viability of Hep1-6 cells in the OD, O, and D groups compared with the control group (Fig. 4F), indicating an enhanced antigen presentation between DCs and CD8^+^ T cells.

To confirm the activation of the cGAS/STING/IFN-I signaling pathway, the protein levels of phosphorylated STING (pSTING) and phosphorylated IRF3 (pIRF3, a downstream marker) were measured by Western blotting in in vitro experiment. Additionally, IFN-β expression was determined by qPCR and ELISA. As shown in Fig. 4G, the expressions of pSTING and pIRF3 in the OD group were higher than in the other group, and the levels in the O and D groups were also higher than in the control groups. Besides, the expression levels of IFN-β in these groups were consistent with those of the pSTING and pIRF3 proteins. Among them, the OD group had the highest expression level (Fig. 4H, I) (*p* < 0.01). Moreover, after inhibiting the expression of STING by a specific inhibitor (c-176), IFN-β levels did not increase obviously compared with the control group (data not shown). On the other hand, the expressions of pSTING, pIRF3, and IFN-β were evaluated in Hep1-6 cells to rule out other potential secretion sources. As expected, they were expressed at a low level comparable to the control group (data not shown).

The expression of IFN-β was also determined in tumor tissues in vivo (Fig. 4J, K). As expected, IFN-β level was significantly up-regulated in the ROD group compared with the other groups (*p* < 0.05). Taken together, these results indicated that the synergistic effect of ROD hydrogel promoted the recruitment and maturation of imDCs, activated the cGAS/STING/IFN-I pathway, enhanced the antitumor immunity, and thus improved the treatment efficacy against residual liver cancer after iRFA therapy.

Moreover, the expressions of IFN-β, pSTING and pIRF3 proteins in DCs were also compared among the groups of lyOK-432, OK-432, LPS, and controls (Additional file 1: Figure S1). The results showed that the mRNA and cytokine levels of IFN-β in the lyOK-432 group were significantly higher than in the OK-432 group (*p* < 0.05), and the protein levels of pSTING and pIRF3 were also obviously higher in the lyOK-432 group when compared with other groups. These results indicated that lyOK-432 also promoted the activation of the cGAS/STING/IFN-I pathway more effectively than non-lysed OK-432.

### Immune memory effects of ROD treatment

**Fig. 5:**
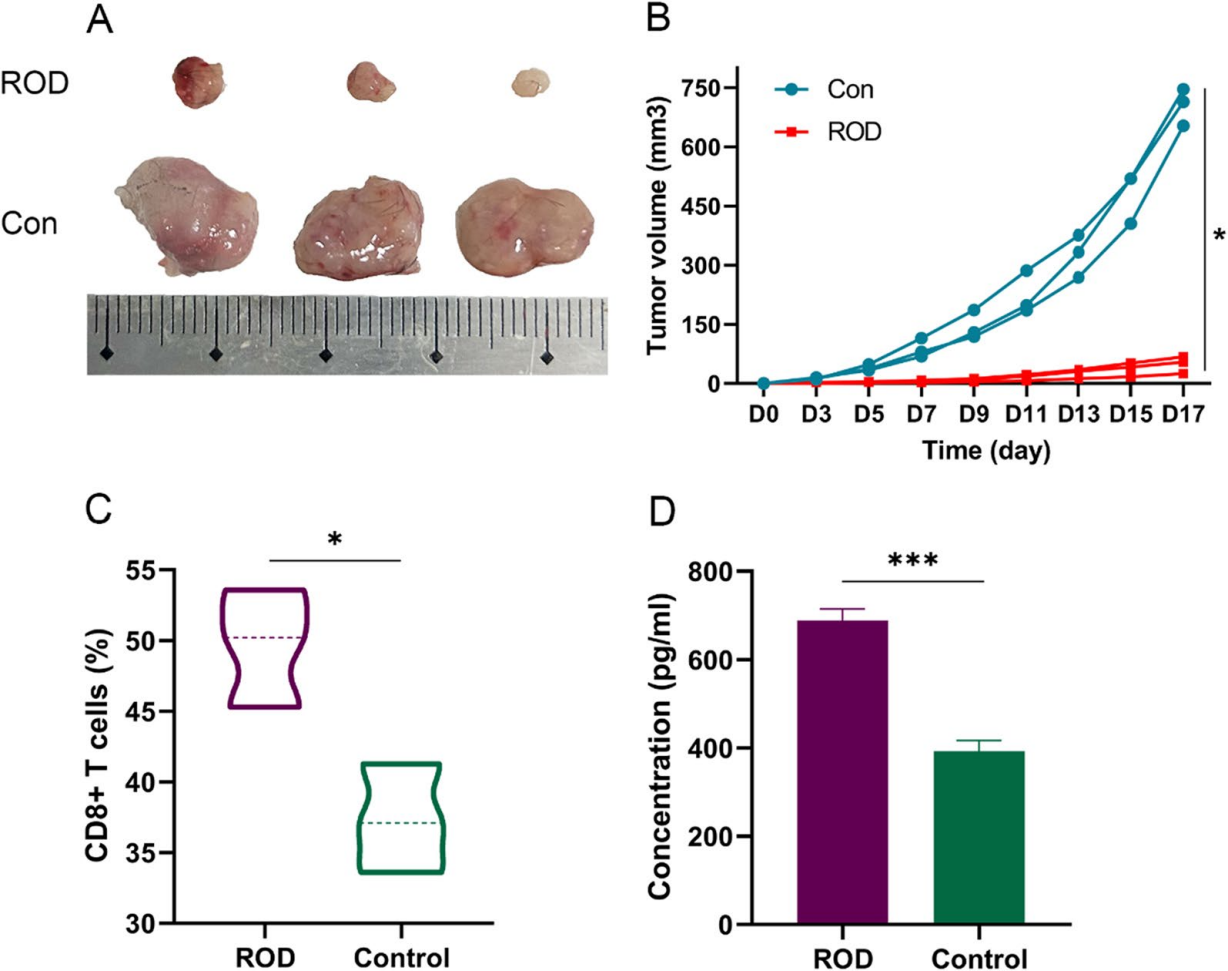
Immune memory effect in the mice that survived after ROD therapy (n = 3). **A** Photograph of dissected tumor samples in the ROD group and blank control group. **B** Measurement of tumor volumes at the indicated times. The subcutaneous tumors were significantly inhibited in ROD group. **C**, **D** Quantitative analysis of CD8^+^ T cells and IFN-γ in the subcutaneous tumors after treatment s, which was significantly higher in ROD group. *p < 0.05; ***p < 0.001

Since the ROD hydrogel treatment presented a potent tumor-inhibitory effect and triggered antitumor immunity, we conducted a re-challenge test to investigate whether an immune memory effect was generated. Mice that survived in the survival experiment (n = 3 in the ROD group) and blank mice (without pre-tumor-bearing, n = 3) were examined. Hep1-6 cells (1 × 10^6^) were subcutaneously injected, and the tumor volume was recorded. As shown in Fig. 5A, B, tumor growth was significantly suppressed in the ROD group (*p* = 0.01), which demonstrated that a strong immune memory effect was generated in these surviving mice.

CD8^+^ T cells and IFN-γ were measured in the tumor tissues of the two groups, and the results suggested that the percentage of CD8^+^ T cells and the expression of IFN-γ were both significantly higher than in the control group (*p* = 0.02 and < 0.001, respectively, Fig. 5C, D).

### Safety evaluation

**Fig. 6:**
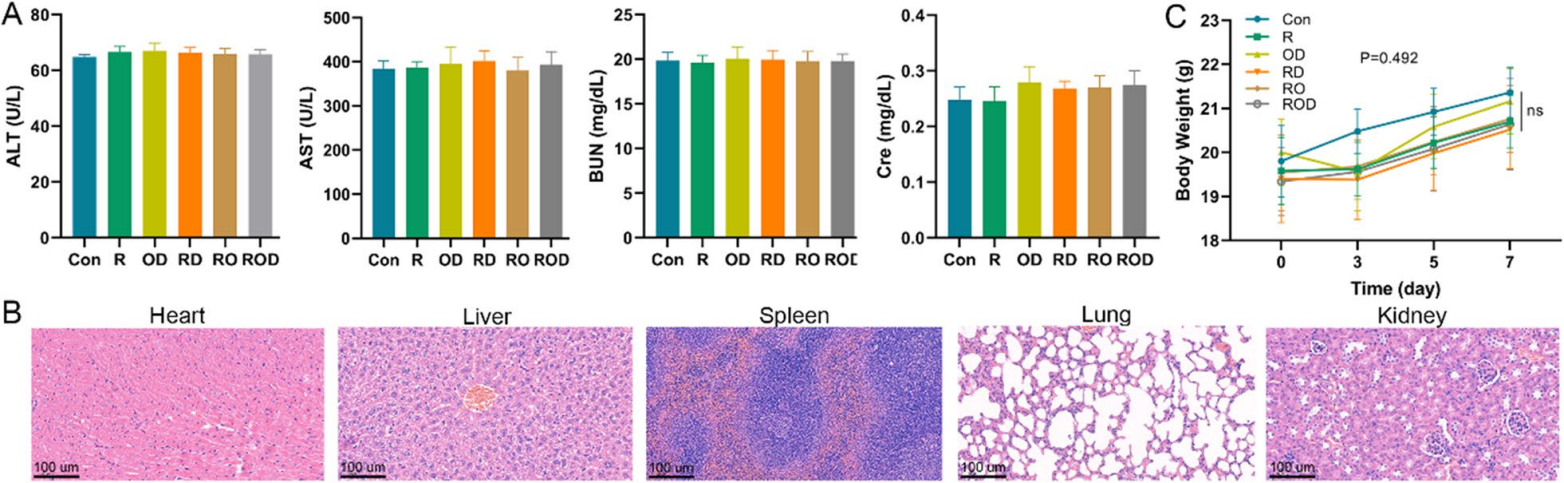
Safety assessment of ROD therapy (n = 5). **A** Liver and renal function were tested 7 days after treatments. **B** HE staining of the heart, liver, spleen, lung, and kidney samples (×20 magnification). **C** Mice body weight at the indicated times after different treatments. These results presented that ROD treatment have no additional damage to mice. *BUN* blood urea nitrogen, *Cre* creatinine, *AST* aspartate aminotransferase, *ALT* alanine aminotransferase. *Ns* not significant

Liver and renal function tests were performed in each group at the indicated time points (Fig. 6). The results suggested that these treatments had little effect on mice liver and renal functions 7 days later. HE staining revealed normal morphologies of major organs, including the heart, liver, spleen, lung, and kidney. The body weight of mice after treatment experienced no significant change compared with the control group (*p* = 0.34). These results indicated that the ROD hydrogel is safe as a treatment for the residual cancer after iRFA of HCC.

## Discussion

Incomplete ablation hampers the widespread application of RFA in the treatment of liver cancer and other malignancies. The aggravated tumor microenvironment after iRFA enhances tumor malignancy, which is difficult to reverse [9]. The present study developed a simple, efficient, and safe ROD hydrogel that substantially improved the tumor-killing efficacy in HCC-bearing mice after iRFA. The synergistic effect of ROD hydrogel treatment recruited more imDCs, promoted their maturation in tumor tissue, activated the intracellular cGAS/STING/IFN-I signaling pathway, enhanced the cytotoxicity of CD8^+^ T cells, increased the proportion of M1-TAMs, reduced the percentage of MDSCs and Treg cells, and finally improved the therapeutic efficacy.

Although OK-432 is widely applied as an immunostimulant for the treatment of cancer, especially in Japan, its efficacy remains to be improved [14, 15]. In this study, OK-432 was lysed before treatment, which had at least two potential advantages. Firstly, lysed OK-432 directly released abundant bacterial peptides, which act as foreign antigens to activate potent innate immunity, including recruitment and maturation of imDCs. Moreover, dsDNA released by lysed OK-432 potentially functioned as a promoter that activates the cGAS/STING/IFN-I pathway in DCs [16]. These advantages were demonstrated in our study by the increased ratios of mDCs, enhanced activation of STING and IRF3 proteins, and elevated expression of IFN-β cytokines in the lyOK-432 group, which has not been reported previously as far as we know.

In recent decades, DOX has been used not only as a routine chemotherapeutic agent for cancers, but also to achieve a certain extent of antitumor immunity. Theoretically, DOX mainly plays the following roles to suppress tumor: (1) It directly kill tumor cells; (2) The dead tumor cells release an “eat me” signal, such as CRT, to help DCs phagocytize apoptotic cells and present tumor antigens; (3) DOX trigger tumor DNA damage, and the DNA fragments activate the cGAS/STING/IFN-I pathway, thus promoting antitumor immunity. To the best of our knowledge, the third mechanism has not been previously investigated in liver cancer, not mention to in combination with OK-432 lysates. Nevertheless, our results demonstrated that DOX could improve the activation of IFN-I pathway in DCs.

Although a previous study indicated that the Toll-like receptor 4 (TLR4) probably implicated in the antitumor immunity of OK-432 when used for the treatment of malignant ascites, the intracellular signaling pathway involved remained unclear [24]. On the other hand, a report demonstrated that the cells regulated by TLR4/TRIF/IRF3 pathway could produce a small amount of IFN-I [25]. However, whether such signaling pathway contributes to the IFN-I production by OK-432 needs further investigation.

However, lyOK-432 or DOX monotherapy is insufficient to destroy tumor [16, 25]. The inadequate treatment efficacy is probably a result of both the tumor burden and the weak antitumor immune response [7]. Yet in this study, combination use of the two agents led to a synergistic therapeutic effect, which could be mechanistically explained by the following factors: (1) The iRFA therapy reduced the most tumor load, substantially easing the burden for the subsequent treatment; (2) The chemotherapeutic effect of DOX further decreased the viable tumor cells, especially after iRFA treatment, which increased the sensitivity of cancer cells to DOX toxicity as we previously found; [26] (3). DOX selectively promoted the proliferation of CD8^+^ T cells and inhibited suppressive immune cells, such as MDSCs and Treg cells, which were increased after iRFA treatment [8, 9, 19]. (4) As foreign antigens, lysed OK-432 recruited and matured the most potent antigen presenting cells, or DCs, into the tumor tissues, which provided the foundation for further antitumor immunity; (5) The thermal ablation and DOX that induced the cell death both promoted the tumor-antigen-presentation of DCs; (6) Self-DNA fragments derived from OK-432 lysates and tumoral DNA resulting from DOX killing both activated the cGAS/STING/IFN-I pathway in DCs. The secreted IFN-β also encouraged the effective antitumor feedback loop [27]. We are led to speculate that the heightened specific antitumor immune responses in this study are the results of all these factors working synergistically.

On the other hand, regular DOX treatment may suppress the immune system [18, 19]. Hence, in the current investigation, a peptide hydrogel was used to induce the controlled-release of DOX and reverse the suppressive role. As previously reported, this hydrogel was an excellent substrate for encasing both hydrophilic and hydrophobic compounds [21]. Our research also showed that the peptide had a satisfactory loading capacity, which also achieved a good controlled-release profile of DOX both in vitro and in vivo. Moreover, the suppressive immune microenvironment was improved by the RD and ROD hydrogels, which indicated the reversed role of standard DOX therapy in immunity after loading DOX in this hydrogel. Additionally, the treatment efficacy of ROD was better than OD in this study, further proving the superiority of this hydrogel.

Previous studies have integrated immune adjuvants with chemotherapeutic agents by means of nanobiotechnology, and accomplished satisfactory treatment efficacy in various cancers [28–30]. For example, Jin et al. proved that K-nanoadjuvant in combination with liposomes (DOX) resulted in a stronger antitumor immunity and better efficacy in murine models compared with K-nanoadjuvant monotherapy [28]. Wang et al. reported that DOX coupled with imidazoquinolines, a TLR7/8 agonist, effectively activated DC and CD8^+^ T cells and improved the therapeutic efficacy in B16-OVA and CT26 tumor-bearing mice models [29]. Chao and his colleagues also demonstrated that the localized cocktail chemoimmunotherapy of DOX/imiquimod/alginate triggers a robust antitumor immune response [30]. Most of the of researches developed their novel materials by using a “pathogen-mimicking” strategy, which take advantages of the size, shape, and surface-molecule organization of pathogens to activate innate and adaptive immunity in cancer therapy. In contrast, in this study, a well-established and safe pathogen, OK-432, was directly loaded, thereby obviating complex processing procedures. Additionally, the intracellular signaling pathway was clarified in our study, which was absent in previous such researches.

As we all known, radiofrequency ablation, DOX and OK-432 all have been clinically and experimentally proved to be safe over past decades. Based on this, lyOK-432 has also been, as expected, shown to be safe, since the dosage and intratumor injection method in our study were theoretically safer than transarterial infusion combined with embolization [31]. Therefore, the ROD treatment has the potential to be put into clinical application.

There was a limitation in this study. The release profile of lysed OK-432 has not been explored, since the components are too complicated to mark.

## Conclusions

This study developed a novel treatment of ROD peptide hydrogel for the liver cancer subjected to iRFA. This synergistic treatment achieved a significant improvement of tumor response and survival benefit in mice, which attributed to the promotion of imDCs recruitment and maturation in tumor tissue, the intracellular activation of cGAS/STING/IFN-I signaling pathway, and the subsequently enhanced antitumor immunity. This treatment modality may lead to a progression in the eradication of larger liver cancer in clinical practice.

### Supplementary Information

**Additional file 1: Figure S1.** LyOK-432 improved DC maturation and activated cGAS/STING/IFN-I pathway. **A**, **B** Measurement of the mature DC surface marker of CD80. C, D Measurement of the mature DC surface marker of CD86. **E**, **F** The cytokines of IL-6 and IL-12 secreted by DCs were measured by ELISA. **G**, **H** The IFN-β expression measured by qPCR and ELISA after management. The expression levels of mature DC surface markers of CD80 and CD86, cytokines of IL-6, IL-12, and IFN-β were significantly higher than other groups. I The protein expression of phosphorylated STING and IRF3 measured by western blotting after management, the activation levels in lyOK-432 group were obvious higher than other groups. **Figure S2.** Effect of the PH on the hydrogel formation and its stability. ROD peptide can gelate at pH 4.5–7.5, while not at pH 8.5. ROD hydrogel was stable for at least 2 weeks. **Figure S3.** The release profile of DOX in RD or ROD hydrogels after intratumor injection in the subcutaneous Hep1-6 tumor model. The fluorescence signals in the two groups were comparable. **Figure S4.** IRFA treatment of the orthotopic Hep 1-6 liver cancer. **A**–**C** Before, during, and after iRFA intervention. D, E MRI images (T2WI and DWI) of the orthotopic Hep 1-6 liver cancer. F HE staining of tumor tissue confirmed the establishment of incompletely tumor ablation model. **Figure S5.** Tumor apoptosis and proliferation measured by TUNEL assay and Ki-67 staining in different groups (×20 magnification). The positive rate of TUNEL staining in ROD group was higher than other groups, while lower of Ki-67 staining. **Figure S6.** Percentages of TAM in different groups. A Flow cytometry analysis of TAMs. B Percentage of CD11b+F4/80+ TAM in total immune cells. C Percentage of CD206+ M2 phenotype in CD11b+F4/80+ TAMs. **Figure S7.** Representative images of immumohistochemical staining of CD4+ T, CD8+ T, Treg cells, and DCs in tumor tissues (×20 magnification).

## Data Availability

The data of this study are available from the corresponding author on reasonable request.

## Abbreviations

RFA: Radiofrequency ablation
lyOK-432: Lysed OK-432
DOX: Doxorubicin
iRFA: Incomplete radiofrequency ablation
DC: Dendritic cell
mDC: Mature DC
ROD: RADA16-I peptide hydrogel loading lyOK-432 and doxorubicin
RO: RADA16-I peptide hydrogel loading lyOK-432
RD: RADA16-I peptide hydrogel loading doxorubicin
R: RADA16-I peptide hydrogel
OD: LyOK-432 combined with doxorubicin
cGAS: Cyclic guanosine phospho-adenosine synthase
STING: Stimulator of interferon genes
IRF3: Interferon regulating factor 3
IFN: Interferon
HCC: Hepatocellular carcinoma
APC: Antigen presenting cell
MRI: Magnetic resonance imaging
DWI: Diffusion weighted imaging
BUN: Blood urea nitrogen
Cre: Creatinine
AST: Aspartate aminotransferase
ALT: Alanine aminotransferase
TIL: Tumor-infiltrating lymphocyte
TAM: Tumor-associated macrophage
MDSCs: Myeloid-derived suppressor cells
