# Correction: Injectable hydrogel loaded with lysed OK-432 and doxorubicin for residual liver cancer after incomplete radiofrequency ablation

**DOI:** 10.1186/s12951-023-02198-2

**Published:** 2023-12-04

**Authors:** Yanyan Cao, Tao Sun, Bo Sun, Guilin Zhang, Jiayun Liu, Bin Liang, Chuansheng Zheng, Xuefeng Kan

**Affiliations:** 1 Department of Radiology, Union Hospital, Tongji Medical College, Huazhong University of Science and Technology, Wuhan, 430022 China; 2 Hubei Province Key Laboratory of Molecular Imaging, Wuhan, China

**Correction: Journal of Nanobiotechnology (2023) 21:404**

10.1186/s12951-023-02170-0

Following publication of the original article [1], the authors identified an error in affiliation 1. The affiliation was incorrectly given as “Department of Radiology, Tongji Medical College, Union Hospital, Huazhong University of Science and Technology, Wuhan 430022, China”, but should have been “Department of Radiology, Union Hospital, Tongji Medical College, Huazhong University of Science and Technology, Wuhan 430022, China”.

This error is corrected in the affiliations list below and the original article [1] has been revised.
