# Supplementary material for: Injectable hydrogel loaded with lysed OK-432 and doxorubicin for residual liver cancer after incomplete radiofrequency ablation
Source: J Nanobiotechnology. 2023 Nov 2;21:404. doi: 10.1186/s12951-023-02170-0 (PMC10623833; doi:10.1186/s12951-023-02170-0)
Supplement: Supplementary file 1 — Additional file 1: Figure S1. LyOK-432 improved DC maturation and activated cGAS/STING/IFN-I pathway. A, B Measurement of the mature DC surface marker of CD80. C, D Measurement of the mature DC surface marker of CD86. E, F The cytokines of IL-6 and IL-12 secreted by DCs were measured by ELISA. G, H The IFN-β expression measured by qPCR and ELISA after management. The expression levels of mature DC surface markers of CD80 and CD86, cytokines of IL-6, IL-12, and IFN-β were significantly higher than other groups. I The protein expression of phosphorylated STING and IRF3 measured by western blotting after management, the activation levels in lyOK-432 group were obvious higher than other groups. Figure S2. Effect of the PH on the hydrogel formation and its stability. ROD peptide can gelate at pH 4.5–7.5, while not at pH 8.5. ROD hydrogel was stable for at least 2 weeks. Figure S3. The release profile of DOX in RD or ROD hydrogels after intratumor injection in the subcutaneous Hep1-6 tumor model. The fluorescence signals in the two groups were comparable. Figure S4. IRFA treatment of the orthotopic Hep 1-6 liver cancer. A–C Before, during, and after iRFA intervention. D, E MRI images (T2WI and DWI) of the orthotopic Hep 1-6 liver cancer. F HE staining of tumor tissue confirmed the establishment of incompletely tumor ablation model. Figure S5. Tumor apoptosis and proliferation measured by TUNEL assay and Ki-67 staining in different groups (×20 magnification). The positive rate of TUNEL staining in ROD group was higher than other groups, while lower of Ki-67 staining. Figure S6. Percentages of TAM in different groups. A Flow cytometry analysis of TAMs. B Percentage of CD11b+F4/80+ TAM in total immune cells. C Percentage of CD206+ M2 phenotype in CD11b+F4/80+ TAMs. Figure S7. Representative images of immumohistochemical staining of CD4+ T, CD8+ T, Treg cells, and DCs in tumor tissues (×20 magnification). [file 12951_2023_2170_MOESM1_ESM.docx]

**Additional file Materials**


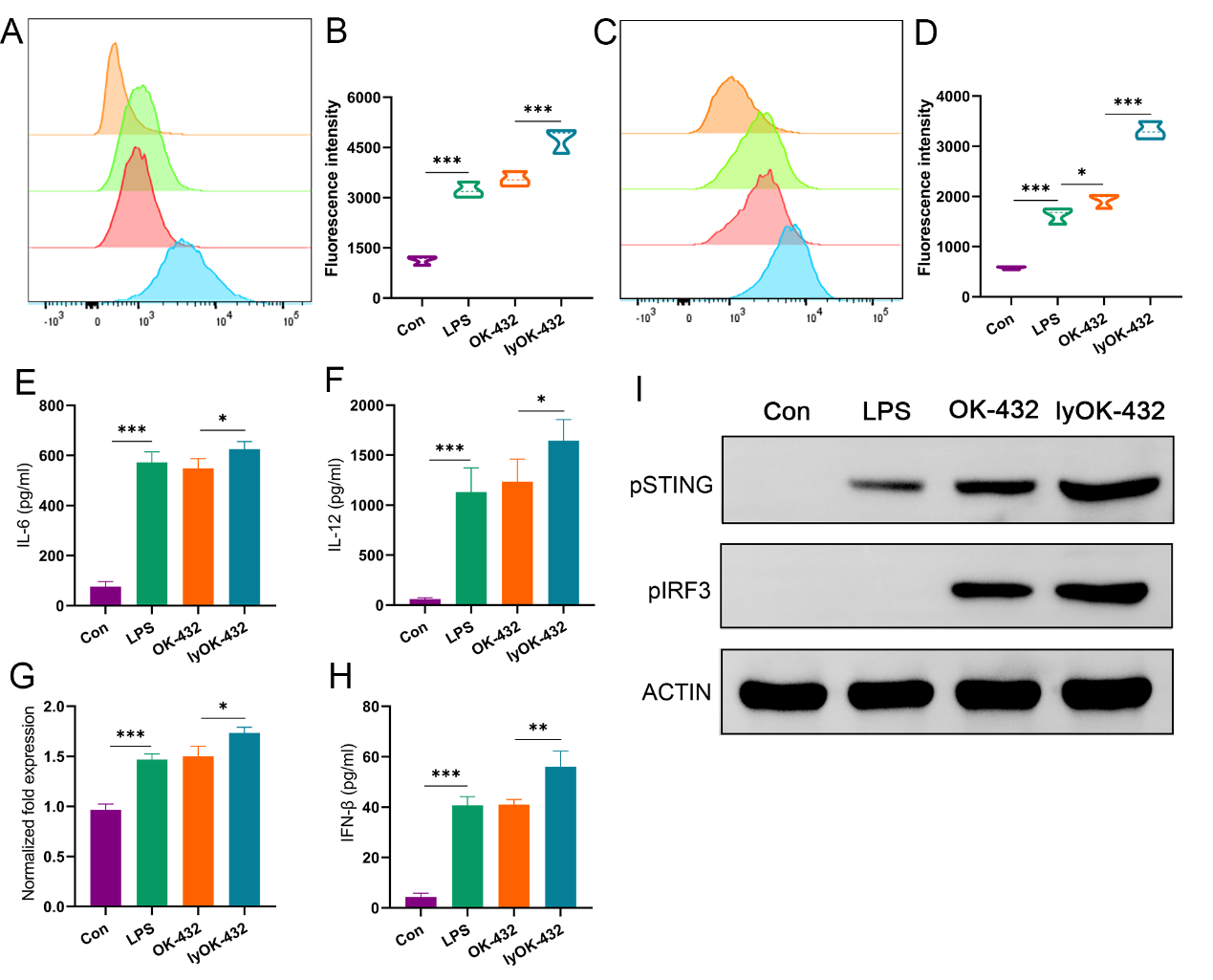


**Figure S1.** LyOK-432 improved DC maturation and activated cGAS/STING/IFN-I pathway. (A, B) Measurement of the mature DC surface marker of CD80. (C, D) Measurement of the mature DC surface marker of CD86. (E, F) The cytokines of IL-6 and IL-12 secreted by DCs were measured by ELISA. (G, H) The IFN-β expression measured by qPCR and ELISA after management. The expression levels of mature DC surface markers of CD80 and CD86, cytokines of IL-6, IL-12, and IFN-β were significantly higher than other groups. (I) The protein expression of phosphorylated STING and IRF3 measured by western blotting after management, the activation levels in lyOK-432 group were obvious higher than other groups.

Con, control; LPS, lipopolysaccharide; lyOK-432, lysed OK-432; DC, dendritic cell; cGAS, cyclic [guanosine](https://www.sciencedirect.com/topics/pharmacology-toxicology-and-pharmaceutical-science/guanosine) phospho-adenosine synthase; STING, stimulator of interferon genes; IRF3, interferon regulating factor 3. *, *p* < 0.05; **, *p* < 0.01; ***, *p* < 0.001.


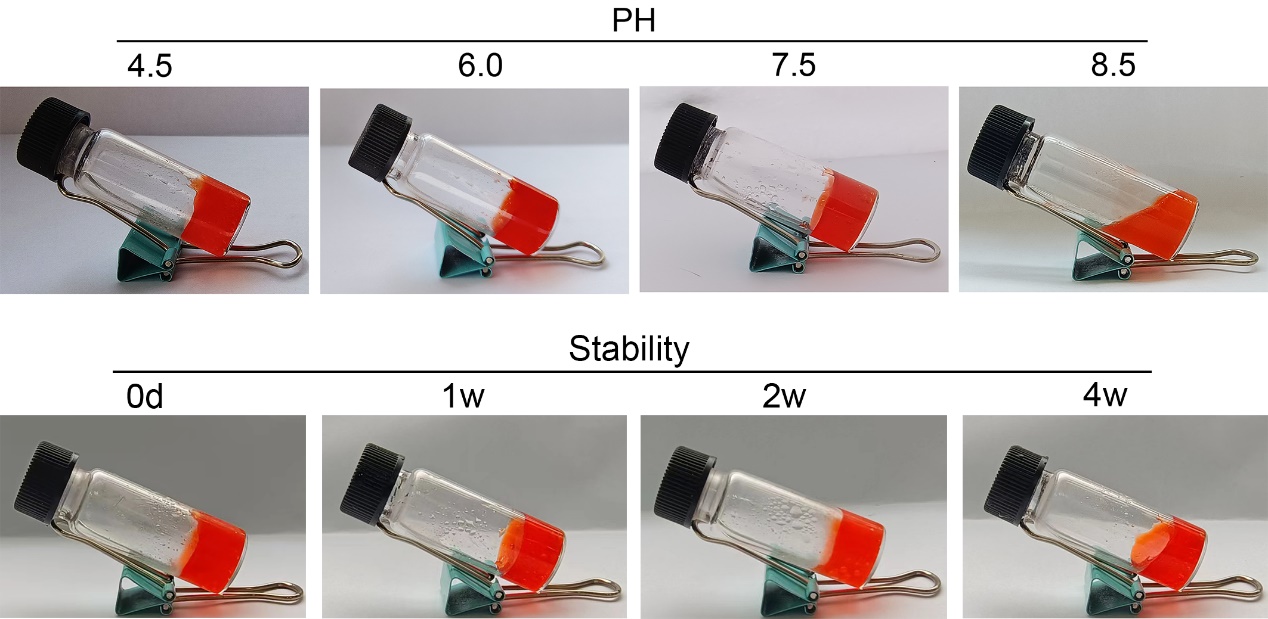


**Figure S2.** Effect of the PH on the hydrogel formation and its stability. ROD peptide can gelate at pH 4.5-7.5, while not at pH 8.5. ROD hydrogel was stable for at least 2 weeks.

RD, RADA16-I peptide hydrogel loaded with doxorubicin.


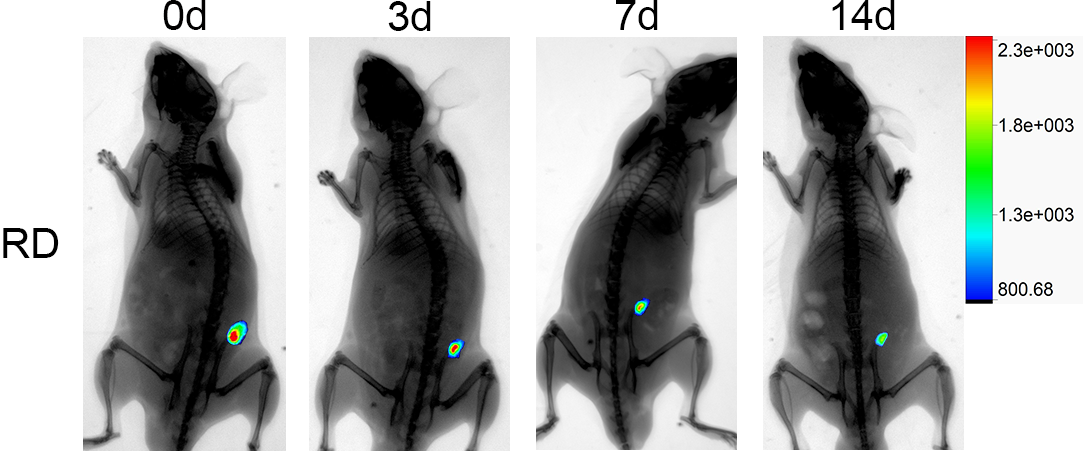


**Figure S3.** The release profile of DOX in RD or ROD hydrogels after intratumor injection in the subcutaneous Hep1-6 tumor model. The fluorescence signals in the two groups were comparable.

DOX, doxorubicin; ROD, RADA16-I peptide hydrogel loaded with lyOK-432 and doxorubicin; RD, RADA16-I peptide hydrogel loaded with doxorubicin.


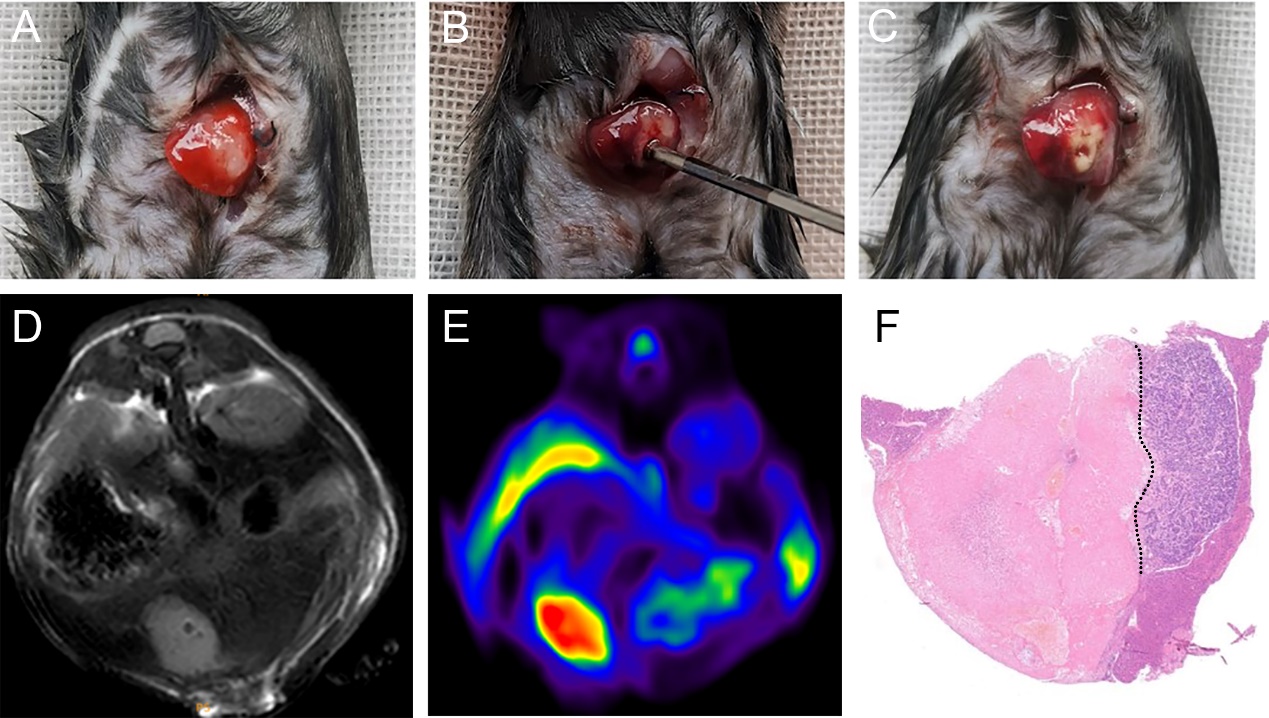


**Figure S4.** IRFA treatment of the orthotopic Hep 1-6 liver cancer. (A-C) Before, during, and after iRFA intervention. (D, E) MRI images (T2WI and DWI) of the orthotopic Hep 1-6 liver cancer. (F) HE staining of tumor tissue confirmed the establishment of incompletely tumor ablation model.

IRFA, incomplete radiofrequency ablation; MRI, magnetic resonance imaging; DWI, diffusion weighted imaging.


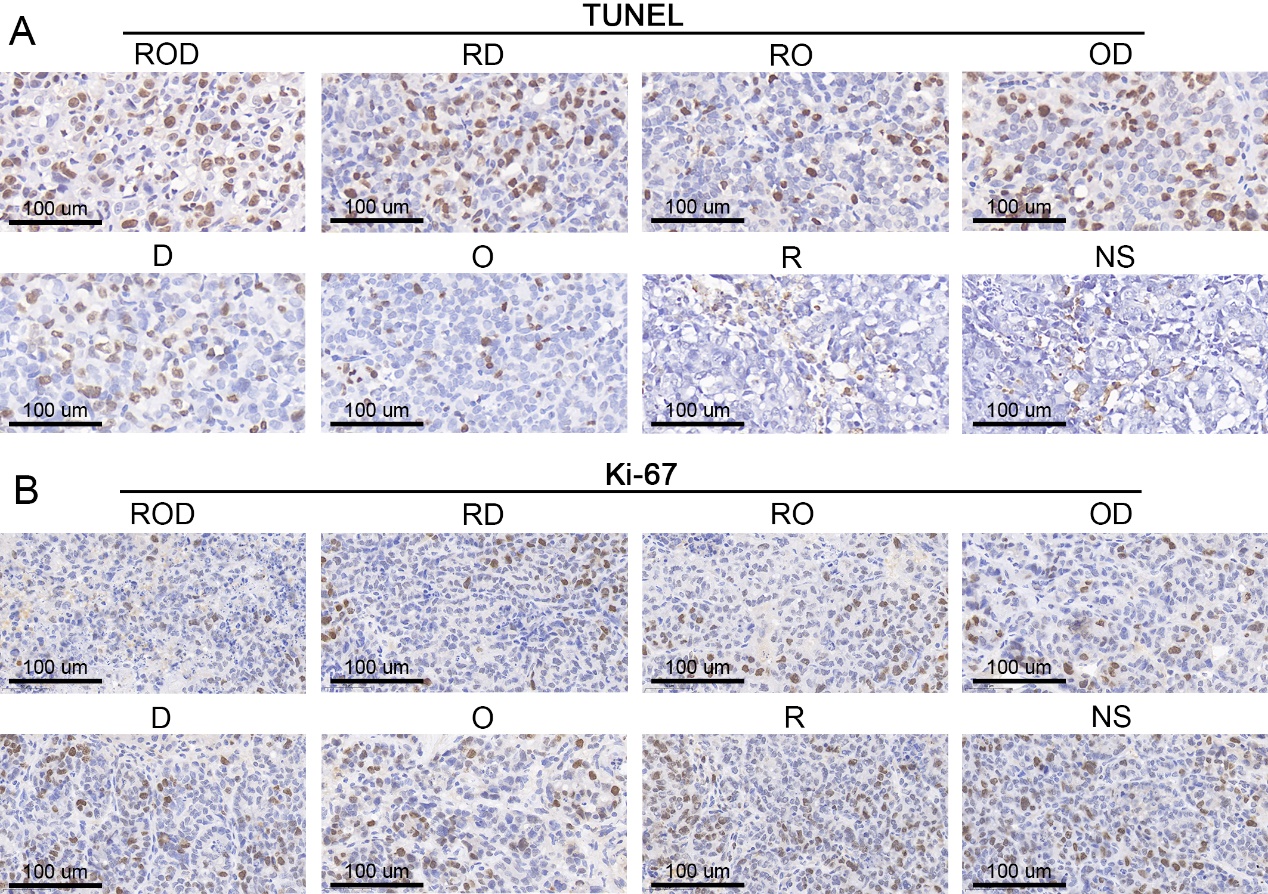


**Figure S5.** Tumor apoptosis and proliferation measured by TUNEL assay and Ki-67 staining in different groups (×20 magnification). The positive rate of TUNEL staining in ROD group was higher than other groups, while lower of Ki-67 staining.

ROD, RADA16-I peptide hydrogel loaded with lyOK-432 and doxorubicin; RO, RADA16-I peptide hydrogel loaded with lyOK-432; RD, RADA16-I peptide hydrogel loaded with doxorubicin; OD, lyOK-432 combined with doxorubicin; D, doxorubicin; O, lyOK-432; R, RADA16-I peptide hydrogel; NS, normal saline.


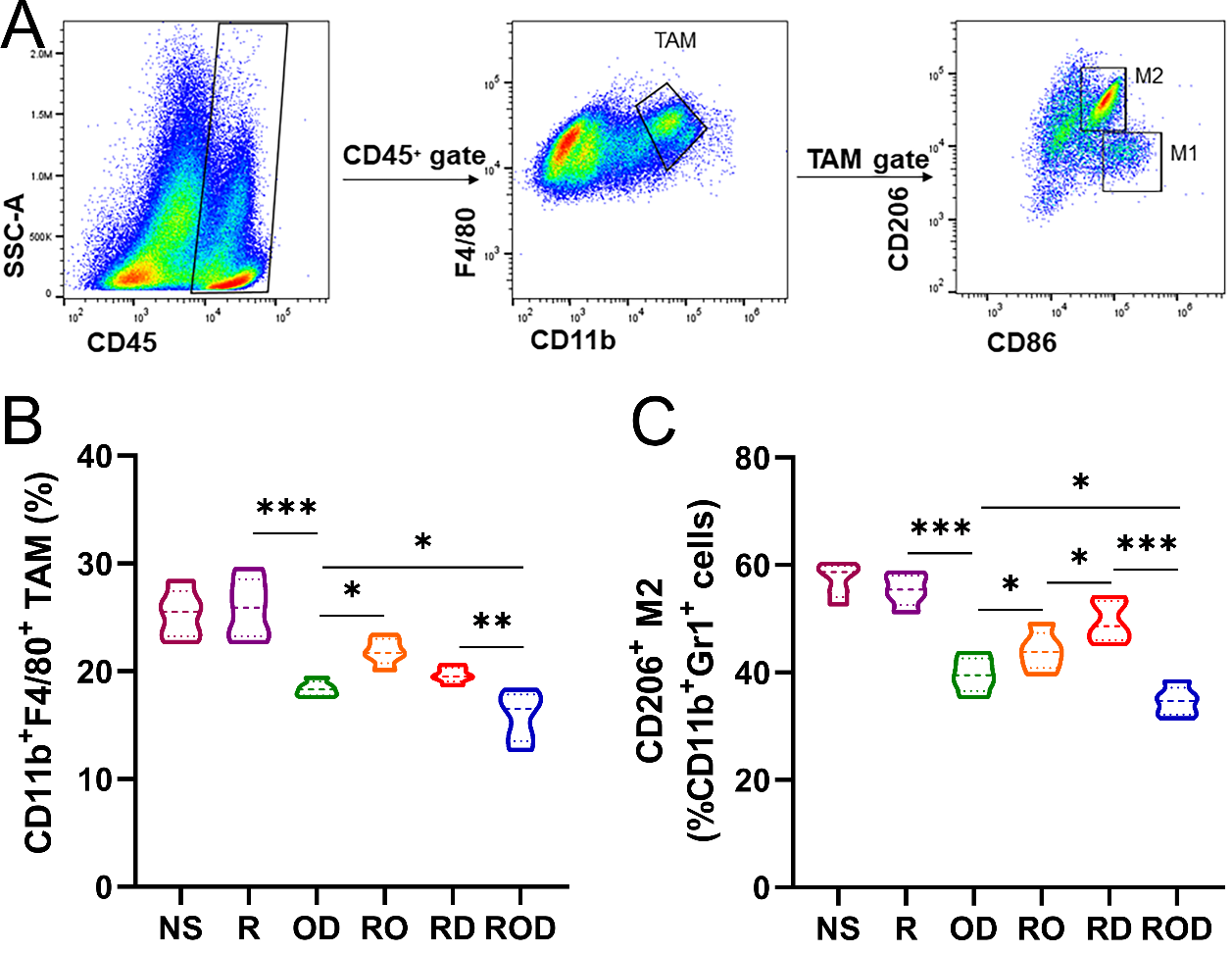


Figure S6. Percentages of TAM in different groups. (A) Flow cytometry analysis of TAMs. (B) Percentage of CD11b^+^F4/80^+^ TAM in total immune cells. (C) Percentage of CD206^+^ M2 phenotype in CD11b^+^F4/80^+^ TAMs.

ROD, RADA16-I peptide hydrogel loaded with lyOK-432 and doxorubicin; RO, RADA16-I peptide hydrogel loaded with lyOK-432; RD, RADA16-I peptide hydrogel loaded with doxorubicin; OD, lyOK-432 combined with doxorubicin; R, RADA16-I peptide hydrogel; NS, normal saline; TAM, tumor-associated macrophage.


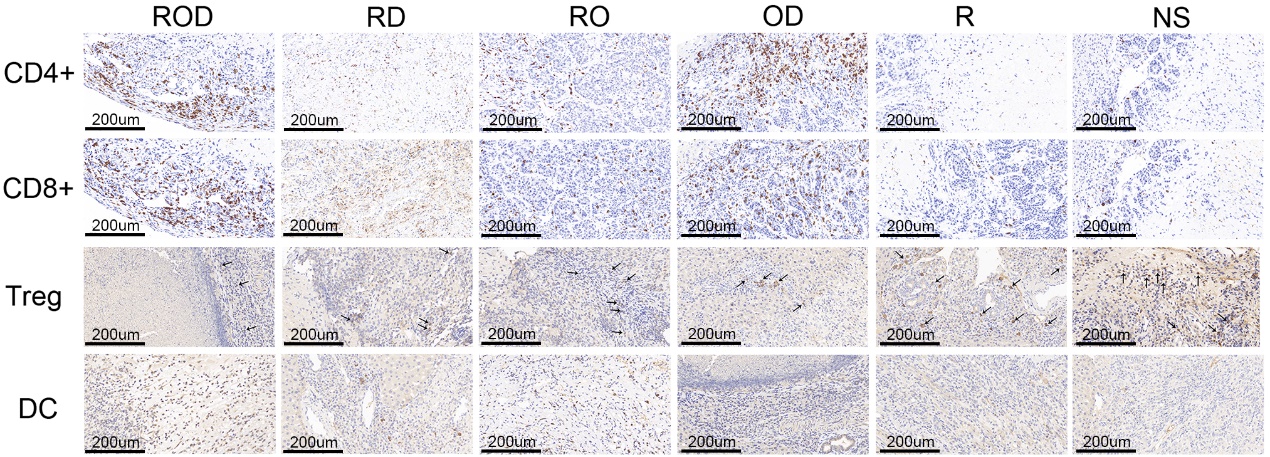


Figure S7. Representative images of [immumohistochemical staining](http://dict.youdao.com/w/immumohistochemical%20staining/#keyfrom=E2Ctranslation) of CD4^+^ T, CD8^+^ T, Treg cells, and DCs in tumor tissues (×20 magnification).

ROD, RADA16-I peptide hydrogel loaded with lyOK-432 and doxorubicin; RO, RADA16-I peptide hydrogel loaded with lyOK-432; RD, RADA16-I peptide hydrogel loaded with doxorubicin; OD, lyOK-432 combined with doxorubicin; R, RADA16-I peptide hydrogel; NS, normal saline.
